# Ascarides

**Published:** 1853-05

**Authors:** C. W. Davis

**Affiliations:** Carlisle, Indiana


ART. V.—*Ascarides.* By C. W. DAVIS, M. D.

MESSRS. EDITORS:—I discover in the February No. of the *North Western Medical and Surgical Journal.* A query made by a correspondent, "for the best remedy for Ascarides."

Several cases laboring under the annoying and troublesome sensation produced by these Worms, having come under my consideration, I will briefly state my treatment, which has generally proved efficient.

I direct the patient to take a large portion of Calomel, Gamboge, and Aloes, in equal proportions,—the quantity to be governed by the age of the patient,—this to be followed in the course of an hour by an enema composed of molasses and sweet milk, or at least to be injected prior to the operation of the Calomel, &c.

After I have succeeded in expelling the Worms, I put the patient upon the use of the Lactate of Iron, or some other chalybeate tonic, to prevent their rapid reproduction.

I took up the idea of the molasses and sweet milk injection from the situation of the worms, believing that none of them were ever discovered, in the different viscera in which the Chyliferous vessels are thickly interspersed, and that Chyle itself was incompatable with their existence.

This purge of Calomel, &c., with the injection, will bring them away frequently in very large quantities, and the Lactate of Iron serves admirably as a preventive against a future annoyance.

Carlisle, Indiana, March, 20, 1853.

